# Qatar’s Journey to Integrating Hospitality Into Healthcare Excellence

**DOI:** 10.7759/cureus.76588

**Published:** 2024-12-29

**Authors:** Justine Ormandy, Megh Mani, Hamad Al-Khalifa, Thabit Melhem, Alanoud H Al-Marri, Mariam N Al-Mutawa, Jibin Kunjavara, Abdulqadir J Nashwan

**Affiliations:** 1 Hospitality Department, Hamad Medical Corporation, Doha, QAT; 2 Health Facility Development, Hamad Medical Corporation, Doha, QAT; 3 Nursing and Midwifery Department, Hamad Medical Corporation, Doha, QAT; 4 Nursing and Midwifery Research Department, Hamad Medical Corporation, Doha, QAT

**Keywords:** healthcare, health quality, hospitality, innovation, world of welcome

## Abstract

The World of Welcome (WOW) program, launched by Hamad Medical Corporation (HMC) in Qatar in 2011, represents a pioneering effort to integrate hospitality principles into healthcare. The program was designed to improve patient and staff experiences in a diverse, multicultural environment. The WOW program was developed to focus on key hospitality values, such as professionalism, empathy, and communication, offering training in multiple languages, including English, Arabic, Hindi, and Malayalam. This approach fostered inclusivity and cultural sensitivity, addressing the unique opportunities of Qatar's demographic composition, where the vast majority of the population comprises expatriates from different cultural and linguistic backgrounds. The initiative expanded under the broader “World of Hospitality” (WOH) umbrella, offering additional programs focused on enhancing patient experience, managing communication, and leadership development. The WOW program has become a model for how healthcare systems, especially in multicultural settings, can integrate hospitality principles to foster a more inclusive, patient-centered, and professional environment, offering valuable insights for healthcare providers globally.

## Editorial

Introduction

In today’s evolving healthcare landscape, there is an increasing recognition of the importance of medical treatment and the overall patient experience. Hamad Medical Corporation (HMC) is the primary provider of secondary and tertiary healthcare in Qatar and one of the leading hospital providers in the Middle East. HMC is leading the development of the region’s first academic health system - combining innovative research, top-class education, and excellent clinical care - and is committed to continue building a legacy of healthcare expertise in Qatar. HMC collaborates with key partners who are experts in Qatar and beyond, including Weill Cornell Medicine-Qatar, the Institute for Healthcare Improvement, and Partners Healthcare, USA [1].

HMC is also the first hospital system in the Middle East to achieve institutional accreditation from ACGME (Accreditation Council for Graduate Medical Education International) International LLC (Limited Liability Company), demonstrating excellence in training medical graduates through residency, internship, and fellowship programs. For more than four decades, HMC has been dedicated to delivering the safest, most effective, and compassionate care to all its patients [2]. As healthcare systems worldwide strive to enhance patient-centered care, Qatar has emerged as a leader through its innovative integration of hospitality into healthcare. HMC launched the “World of Welcome” (WOW) program in 2011, a transformative initiative emphasizing hospitality as an essential element of patient care. Qatar’s approach, however, is distinct in its ability to cater to a diverse workforce and patient population, reflecting the nation’s unique demographic makeup.

Qatar is home to a highly multicultural society, with a significant portion of its workforce being expatriates. Over 85% of the country’s population comprises expatriates worldwide, creating a complex and diverse healthcare environment. This diversity presents challenges and opportunities for healthcare delivery, as employees and patients come from various cultural and linguistic backgrounds. The WOW program provides training in multiple languages, including English, Arabic, Hindi, and Malayalam. It has been a critical tool for uniting HMC’s workforce under a shared professionalism and patient-centered care ethos. This editorial explores Qatar’s journey in integrating hospitality into healthcare and its global implications for diverse healthcare systems [3].

Enhancing the patient’s experience is an ongoing challenge that evolves with shifting patient expectations and growing competition in healthcare. Both clinical and non-clinical staff play essential, although distinct, roles in patient-centered care: clinical teams provide skilled, compassionate medical attention, while non-clinical staff manage the logistical and environmental factors contributing to a smooth, welcoming experience. Using hospitality best practices, healthcare providers aim to create a functional, comfortable, and reassuring environment. This focus on hospitality is particularly significant, as patients typically turn to healthcare providers like HMC out of need rather than choice. By prioritizing empathy, clear communication, and seamless processes, healthcare institutions can help ease the anxiety often associated with medical care, enhancing trust and fostering a holistic, positive patient experience that resonates well beyond the walls of the clinic or hospital. The staff and patients at HMC come from diverse cultural and linguistic backgrounds, necessitating an approach that addresses these differences while fostering unity and professionalism [4].

The WOW program at HMC was developed to bridge cultural and linguistic gaps within its diverse workforce while fostering a unified understanding of hospitality in patient care [5]. By providing training in multiple languages and emphasizing core principles of professionalism, communication, and empathy, the program aims to create a welcoming healthcare environment. Its primary goals include improving the overall patient experience by integrating hospitality into healthcare delivery, enhancing staff engagement, and promoting teamwork among culturally diverse employees to ensure better communication and higher patient satisfaction.

The inception of the “World of Welcome” program

In 2011, the WOW program was born out of this realization, targeting employees at every level of the organization, from senior executives to support staff [6]. By attending the WOW program, participants learn about the core values of hospitality - Fresh, Host, and Easy - and how these principles apply to patient care. Through practical examples and interactive learning, staff are equipped with the tools to improve communication, foster teamwork, and enhance the patient experience [6].

By 2014, the program had reached over 10,000 staff members, including clinical and non-clinical personnel from various hospitals and departments. With a satisfaction rate of 94% (post-program survey), it was clear that the WOW program was addressing the practical needs of HMC’s workforce and fostering a sense of unity and shared purpose among staff from different cultural backgrounds (Table 1).

**Table 1:** Annual participation in hospitality programs in the year 2011-2024 WOW: World of Welcome, WOS: World of Service, WOO: World of Opportunity, WOC: World of Communication, TLL: Take the Lead

Hospitality programs	2011-2014	2015	2016	2017	2018	2019	2020	2021	2022	2023-2024	Total
WOW	10850	2440	2262	3050	3219	3274	998	770	1881	3735	32479
WOC	725	1507	1411	1409	1562	1107	448	396	182	786	9533
WOO	434	1274	1103	1133	1383	1071	574	463	296	679	8410
WOS	806	1633	1311	1269	1665	1160	529	426	115	709	9623
TTL	516	283	328	248	233	240	101	54	145	335	2483
Total	13331	7137	6385	7109	8062	6852	2650	2109	2619	6244	62528

**Figure 1:**
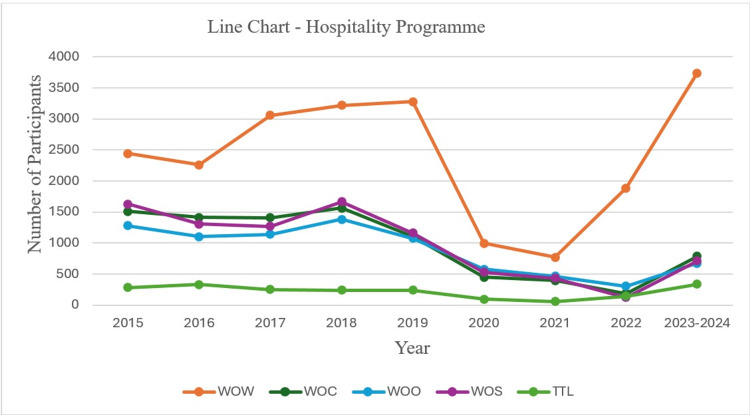
Line chart showing participant trends in hospitality programs for 2015 to 2024. WOW: World of Welcome, WOS: World of Service, WOO: World of Opportunity, WOC: World of Communication, TLL: Take the Lead

The line chart illustrates participant trends from 2015 to 2024 across different segments of a Hospitality Programme: WOW (World of Welcome), WOS (World of Service), WOO (World of Opportunity), WOC (World of Communication), and TTL (Take the Lead). WOW, shown in orange, is the most dynamic, starting with about 2000 participants in 2015, climbing to around 3000 by 2018-2019, then experiencing a sharp decline to nearly zero in 2020. However, WOW rebounded significantly, peaking at around 3500 participants in 2023-2024. WOC (green) aligns more with the WOS and WOO in terms of Participants throughout the year. WOO (blue) and WOS (purple) start with around 1500 participants, showing slight fluctuations and a dip in 2021 but exhibiting a gradual recovery towards the end of the period. TTL (light green), representing the total number of participants, remains relatively stable but shows a slight uptick in 2023-2024, primarily influenced by WOW’s resurgence. This chart underscores WOW’s major impact on the total trend, with other segments contributing steadily but smaller participants (Figure 1).

Qatar’s vision: a diverse workforce, a unified approach

Qatar’s healthcare system, like its broader economy, is powered by a diverse, expatriate workforce. With healthcare workers hailing from countries as far-ranging as India, the Philippines, Egypt, and the United Kingdom, creating a unified approach to patient care is both a challenge and a necessity. The WOW program’s success lies in its ability to transcend cultural barriers by offering a framework that all staff, regardless of their background, can relate to and apply.

This approach aligns with Qatar’s vision of becoming a global leader in healthcare delivery. Qatar has invested heavily in healthcare infrastructure and innovation as one of the wealthiest and fastest-growing nations in the Middle East. But the human element remains at the core of its healthcare strategy. The WOW program is a testament to Qatar’s commitment to creating a welcoming, professional environment for patients and staff, which values cultural diversity and leverages it to enhance care [7].

Healthcare systems globally face similar challenges when dealing with diverse populations, whether in the multicultural cities of the United States, the United Kingdom, or Australia. The WOW program offers a valuable model for navigating these challenges, showing that hospitality principles can create a cohesive and effective workforce, even in the most diverse settings [8].

Expanding the Hospitality Model

**Figure 2:**
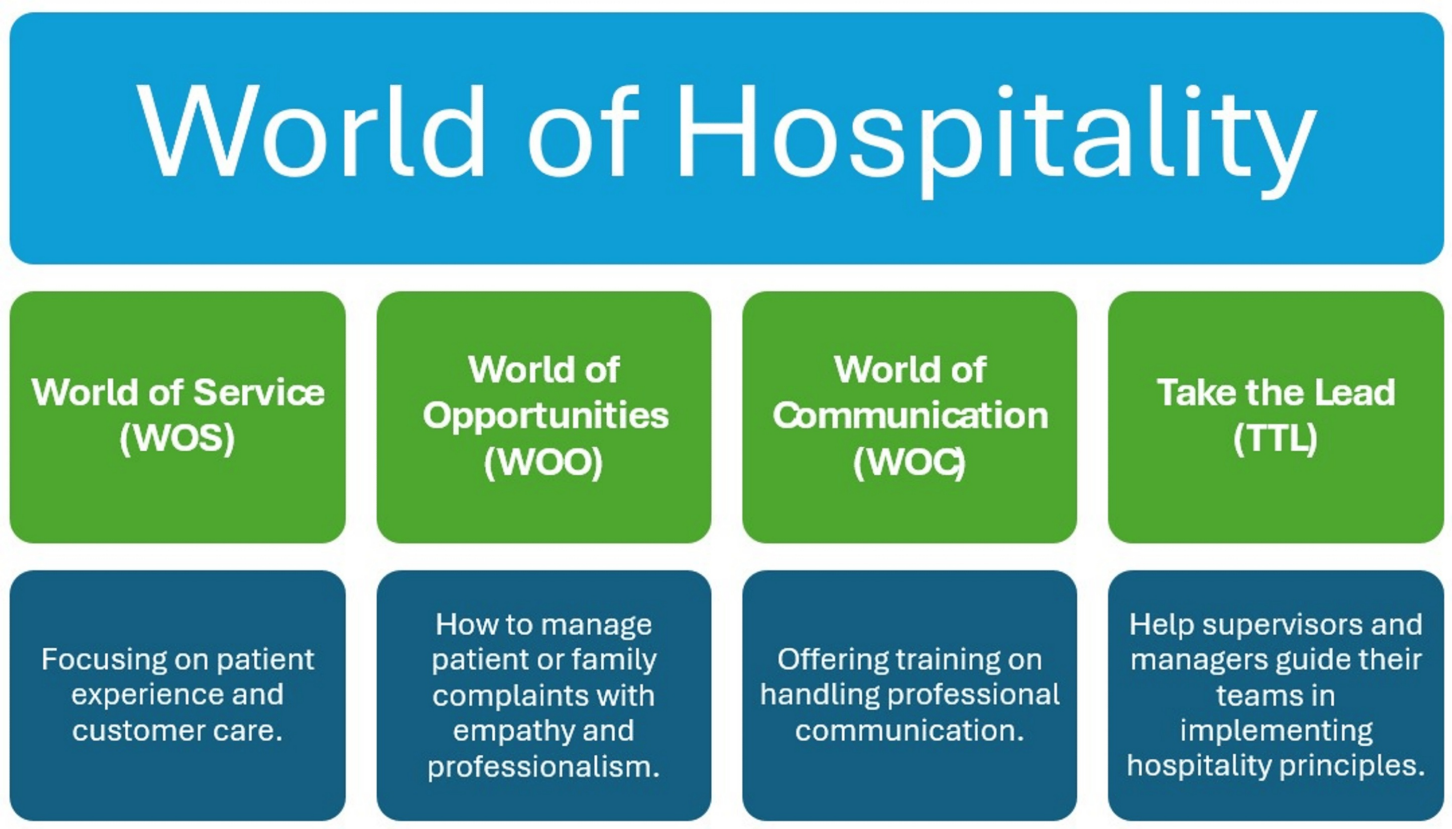
World of Hospitality program in Qatar.

Following the success of the WOW program, Qatar continued to expand its hospitality training model. By 2017, HMC had introduced several complementary programs under the “World of Hospitality” (WOH) umbrella (Figure 2).

By the end of 2017, more than 32,000 staff members, including many expatriates from HMC’s hospitals and corporate departments, had participated in the WOH programs [9]. The overall satisfaction rate remained high at 93%, with 98% of participants recommending the courses to others. These programs significantly impacted workplace culture, fostering better communication and teamwork among employees from different nationalities.

Hospitality in Healthcare: A Global Movement

Qatar’s WOW program has far-reaching implications for healthcare systems globally, especially in countries with similarly diverse workforces. Hospitals and healthcare providers increasingly focus on patient experience as a key performance indicator in many parts of the world. The principles of hospitality professionalism, empathy, and effective communication are recognized as vital components of high-quality healthcare delivery [10].

In multicultural cities like New York, London, and Sydney, healthcare providers are grappling with the challenges of serving diverse populations while maintaining the highest standards of care. Qatar’s approach offers a valuable lesson: training incorporating cultural and linguistic diversity can help foster a more inclusive and harmonious workplace. This, in turn, leads to better patient outcomes (e.g., improved patient experience), as healthcare providers are better equipped to address the needs of patients from various backgrounds [11].

Adapting to New Challenges: The Impact of COVID-19

The COVID-19 pandemic introduced new challenges to healthcare systems worldwide, including Qatar’s. In response to the pandemic, HMC adapted its WOW and other WOH programs by transitioning to online learning formats. Virtual courses on platforms like Microsoft Teams allowed the programs to continue, ensuring that new and existing staff could complete their training despite the restrictions on face-to-face interactions.

This shift to digital learning also reflected Qatar’s agility in responding to global healthcare challenges. The WOW program’s ability to adapt to online delivery demonstrates how hospitality training can be sustained even in the face of unprecedented crises, offering a model for healthcare systems worldwide that are still navigating the impact of the pandemic [11,12].

The Road Ahead: Embracing Diversity in Healthcare

As Qatar’s healthcare system continues to grow and evolve, integrating hospitality will remain an essential element of its strategy. The WOW program’s success in uniting a diverse expatriate workforce under a shared vision of patient-centered care is a testament to Qatar’s leadership in healthcare innovation. Recognizing and valuing the culture of its employees' cultural diversity creates healthcare where patients and staff feel welcomed, respected, and empowered.

Globally, healthcare systems are increasingly looking to models like Qatar’s WOW program to enhance patient experience and improve staff engagement. By emphasizing hospitality, Qatar has set a standard for healthcare systems worldwide to follow, demonstrating that diversity can be a strength rather than a challenge.

Recommendations

To advance Qatar’s WOW program, the focus should be optimizing digital learning, enhancing cultural competency training, and leveraging diversity to improve patient-centered care. Comprehensive research using mixed methods to evaluate key metrics like patient satisfaction and staff engagement, benchmarking, and longitudinal studies will provide insights into its effectiveness and sustainability. By refining the program through global collaboration and extending its principles to community health services, WOW can be a replicable model, inspiring healthcare systems worldwide while preparing for future challenges.

Conclusion

In conclusion, this editorial outlines the WOW program's foundational vision and preliminary participation data, emphasizing its role in fostering a unified understanding of hospitality within a culturally diverse workforce. While the program has demonstrated promising engagement, assessing its long-term impact on healthcare quality, workforce satisfaction, and patient experience requires further investigation. Future studies should focus on evaluating the program's quality, exploring its influence on optimizing the health system, and identifying key factors driving systemic change. In addition, comprehensive employee and patient experience assessments are necessary to fully understand the program’s transformative potential and provide a robust framework for its implementation in other healthcare settings.

## References

[1] (2024). Hamad Medical Corporation. https://www.hamad.qa/EN/Pages/default.aspx.

[2] Schwartz A (2001). Assertiveness: responsible communication. https://books.google.com.ph/books/about/Assertiveness.html?id=rgSGZdr56VkC&redir_esc=y.

[3] (2024). Assertiveness - tips & techniques. https://www.skillsyouneed.com/ps/assertiveness-techniques.html.

[4] Ghanim Al Muftah (2024). Ghanim Al Muftah: contact. https://ghanimalmuftah.org/.

[5] (2024). Defining patient experience - The Beryl Institute. https://theberylinstitute.org/defining-patient-experience/.

[6] (2024). Improving patient experience: patient-centric admissions at St. John of God Health Care. https://www.pwc.com/gx/en/about/case-studies/st-johns.html.

[7] Huang L, Zhang J, Huang Q, Cui R, Chen J (2023). In-hospital major adverse cardiovascular events after primary percutaneous coronary intervention in patients with acute ST-segment elevation myocardial infarction: a retrospective study under the China chest pain center (standard center) treatment system. BMC Cardiovasc Disord.

[8] (2024). Itawasol Home. https://itawasol.hamad.qa/EN/Pages/default.aspx.

[9] Oben P (2020). Understanding the patient experience: a conceptual framework. J Patient Exp.

[10] (2024). Patient Experience Journal - research and proven practices on patient experience excellence and human experience in healthcare. The Beryl Institute. https://pxjournal.org/journal/.

[11] (2024). Sentinel Event Data Summary. The Joint Commission. https://www.jointcommission.org/resources/sentinel-event/sentinel-event-data-summary/.

[12] Asia HM. (2024). The Importance of clinical communication and collaboration in healthcare. HMA.

